# Glucocorticoids ameliorate TGF-β1-mediated epithelial-to-mesenchymal transition of airway epithelium through MAPK and Snail/Slug signaling pathways

**DOI:** 10.1038/s41598-017-02358-z

**Published:** 2017-06-14

**Authors:** Hyun-Woo Yang, Seoung-Ae Lee, Jae-Min Shin, Il-Ho Park, Heung-Man Lee

**Affiliations:** 1 Department of Biomedical Science, Korea University, College of Medicine, Seoul, Korea; 2 Institute for Medical Devices Clinical Trial Center, Korea University Guro Hospital, Korea University, College of Medicine, Seoul, Korea; 3 Department of Otorhinolaryngology-Head and Neck Surgery, Korea University, College of Medicine, Seoul, Korea; 4 Research-Driven Hospital, Korea University Guro Hospital, Korea University, College of Medicine, Seoul, South Korea

## Abstract

Chronic rhinosinusitis with nasal polyps (CRSwNP) is closely associated with tissue remodeling. Epithelial-to-mesenchymal transition (EMT), a process of tissue remodeling, can be a therapeutic target of CRSwNP. Glucocorticoids are a type of steroid hormone that is used primarily in medical therapy for patients with CRSwNP; however, their effects on EMT in the airway epithelium remain unknown. To investigate the effects of dexamethasone and fluticasone propionate, a class of glucocorticoids, on transforming growth factor-β1 (TGF-β1) -induced EMT, we used A549 cells, human primary nasal epithelial cells (hPNECs) and *ex vivo* organ culture of the inferior turbinate. TGF-β1 induced changes in cell morphology, suppressed the expression of E-cadherin and enhanced the expression of a-smooth muscle actin, vimentin and fibronectin in A549 cells. However, glucocorticoids inhibited EMT, migration and invasion enhancement by TGF-β1. We found that the induction of phosphorylated ERK, p38 and the activity of Snail and Slug transcription factors by TGF-β1 were suppressed by glucocorticoids. Glucocorticoids also had a similar effect in hPNECs and *ex vivo* organ cultures of the inferior turbinate. These findings suggest that glucocorticoids might be a useful therapy for preventing tissue remodeling by blocking the EMT initiated by TGF-β1-induced MAPK and Snail/Slug signaling pathways in CRSwNP.

## Introduction

Chronic rhinosinusitis (CRS) is a multifactorial inflammatory disease that occurs in paranasal sinuses and accompanies symptoms such as nasal obstruction, nasal airway inflammation and nasal discharge lasting for more than 12 weeks. There are two types of CRS: CRS without nasal polyps (CRSsNP) and CRS with nasal polyps (CRSwNP), which are heterogeneous. CRSwNP is characterized by the presence of CRS in the paranasal sinuses and nasal polyp growth in the upper nasal cavities^1^. Nasal polyps, a type of benign sinonasal tumor, are noncancerous abnormal growths inside the nose. They tend to be slow growing and do not metastasize to other parts of the body^2^. Larger polyps that cause problems with the ability to breathe or sense of smell and vision can be treated surgically or medically. Even though complete surgical removal is the best available treatment^3, 4^, a precise understanding of the mechanism underlying nasal polyp formation is required to develop therapies that target the molecules involved in these physiological and biological changes.

Tissue remodeling is a reconstruction process for wound repair, but can cause pathological reconstruction such as changes in the airway epithelium, lamina propria and submucosal regions in a variety of chronic airway diseases including CRS^5^. The epithelial-to-mesenchymal transition (EMT) is considered a critical process involving embryonic development and the progression of carcinoma^6^. It has been observed in CRS and is a process of tissue remodeling, which induces loss of epithelium and differentiation to myofibroblasts. As a result, the epithelium loses its protective role against external antigens, and studies have found that this phenomenon is associated with the pathogenesis of nasal polyps *in vivo* and in patients with CRS^7–9^.

We previously reported that transforming growth factor-β1 (TGF-β1), a representative profibrotic cytokine, initiates tissue remodeling in the airway epithelium through activation of EMT signals^10^. During the EMT progression, the cell-cell adhesion and polarity of the epithelial cells gradually decrease, and epithelial cells change into mesenchymal cells. This change leads to development of a spindle-shaped morphology and characteristics such as migration, invasion, and wound healing^11, 12^. EMT is based on the down-regulation of epithelial markers such as E-cadherin and the up-regulation of mesenchymal markers such as alpha-smooth muscle actin (α-SMA), vimentin and fibronectin^13, 14^. Transcription factors such as Snail and Slug, zinc-finger transcriptional repressors, regulate E-cadherin expression by binding E-cadherin promoters (E-boxes), which are influenced by EMT events^15, 16^.

Dexamethasone and fluticasone propionate, which are synthetic glucocorticoids (GCs), possess potent anti-inflammatory and immunosuppressive properties and are used to treat asthma, allergic rhinitis, nasal polyps, and skin disorders^17^. Dexamethasone treatment suppresses EMT-related morphological changes and protein alteration through Smad and non-Smad signaling pathways in hepatocytes^18^. An inhaled fluticasone propionate clinical trial found evidence of a link between EMT and epithelial cell activation in patients with chronic obstructive pulmonary disease^19^. However, the impact of GC treatment on airway tissue remodeling is controversial and remains unclear.

In this study, we investigated the effects of GCs on TGF-β1-induced EMT in the airway epithelium.

## Results

### Glucocorticoids suppressed TGF-β1-induced EMT in A549 cells

**Figure 1:**
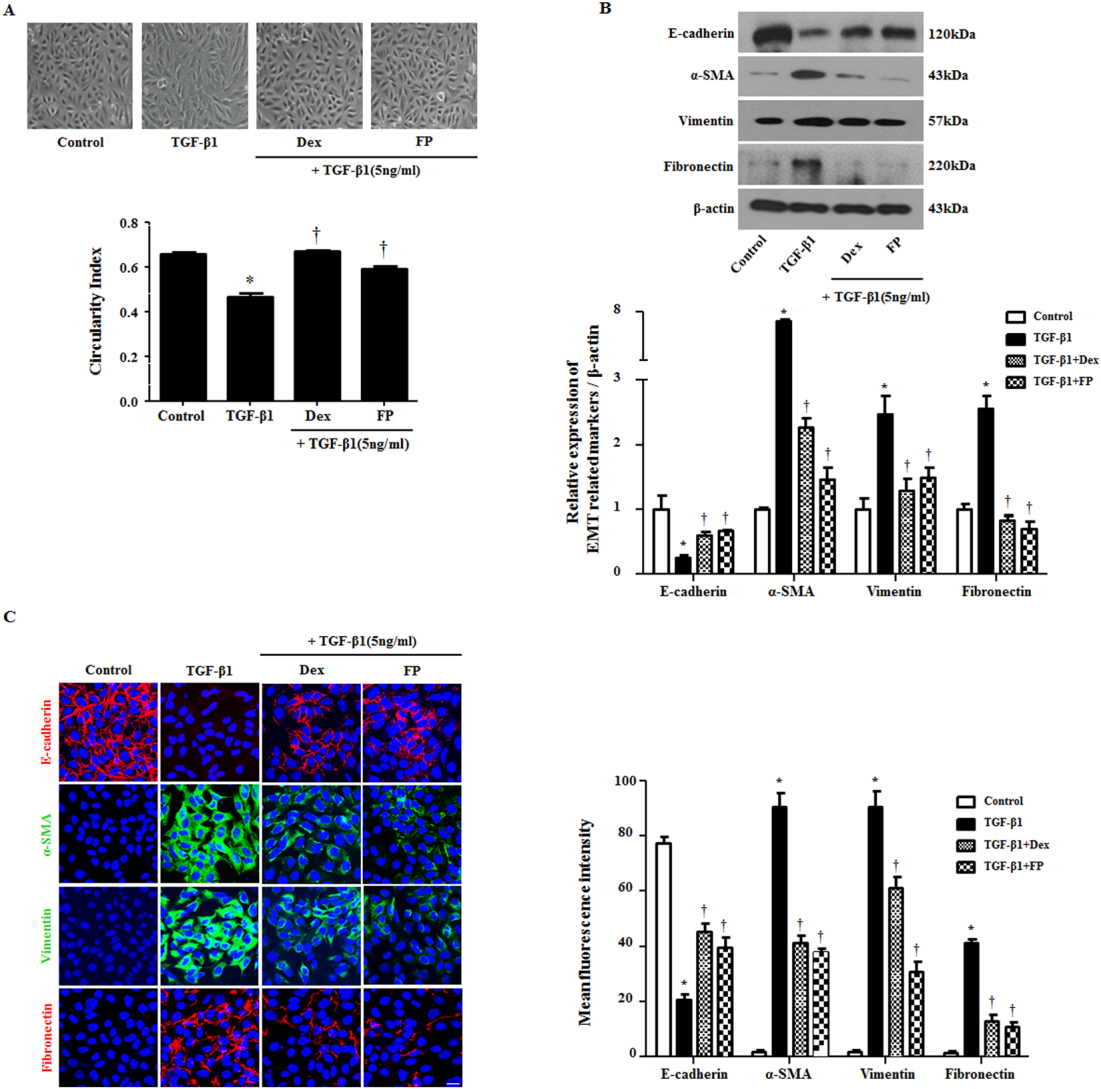
Glucocorticoids suppress EMT-related alterations by TGF-β1 in A549 cells. Cells were pretreated with or without dexamethasone (Dex, 2.5 μM) and fluticasone propionate (FP, 2.5 μM) for 1 hour and then stimulated with TGF-β1 (5 ng/ml) for 72 hours. (**A**) Cell morphology images were acquired by phase contrast microscopy and cell circularity was calculated using ImageJ software Expression of EMT-related markers was measured by (**B**) western blots and (**C**) immunofluorescence. Immunofluorescence images were captured by confocal laser scanning microscope. Western blot data were normalized to β-actin. Representative fluorescein immunocytochemical staining showed E-cadherin (red), α-SMA (green), vimentin (green) and fibronectin (red) with nuclear DAPI (blue). Scale bar = 20 μm. All data are presented as means ± SEM. Results were from at least three independent experiments. *p < 0.05 vs. control; ^†^p < 0.05 vs. TGF-β1.

To determine whether GCs suppressed TGF-β1-induced EMT in A549 cells, we first evaluated the cytotoxic effect of GCs on A549 cells using MTT assays after treatment with dexamethasone or fluticasone propionate at the indicated doses (0–80 μM) for 72 hours. Given that we previously found that TGF-β1 induces EMT in the airway epithelium with mesenchymal features^10^, we sought to determine the effects of GCs on TGF-β1-induced EMT in A549 cells. Cells were pretreated with dexamethasone (2.5 μM) or fluticasone propionate (2.5 μM) for 1 hour and then stimulated with TGF-β1 (5 ng/ml) for 72 hours. Phase contrast images revealed that treatment with TGF-β1 changed cells from a typical cobblestone-shape to elongated, flattened and spindle-like shapes. GCs significantly ameliorated TGF-β1-induced morphological changes, which were quantitatively determined by cell circularity (p < 0.05, Fig. 1A). Consistent with the observed morphological changes, TGF-β1 treatment decreased the expression of epithelial marker E-cadherin, however increased mesenchymal markers α-SMA, vimentin, and fibronectin. In the groups pretreated with GCs, we observed the recovery of E-cadherin expression and loss of α-SMA, vimentin and fibronectin expression (Fig. 1B). Immunofluorescence indicated that TGF-β1 treatment reduced E-cadherin expression in the membrane and induced α-SMA, vimentin and fibronectin expression in the cytoplasm; this effect was rescued by dexamethasone and fluticasone propionate, suggesting that GCs reduced TGF-β1-mediated EMT processes in airway epithelial cells, leading to the alteration of EMT-related protein expression and to changes in cellular morphology (Fig. 1C).

### Glucocorticoids inhibited TGF-β1-induced migration of A549 cells

**Figure 2:**
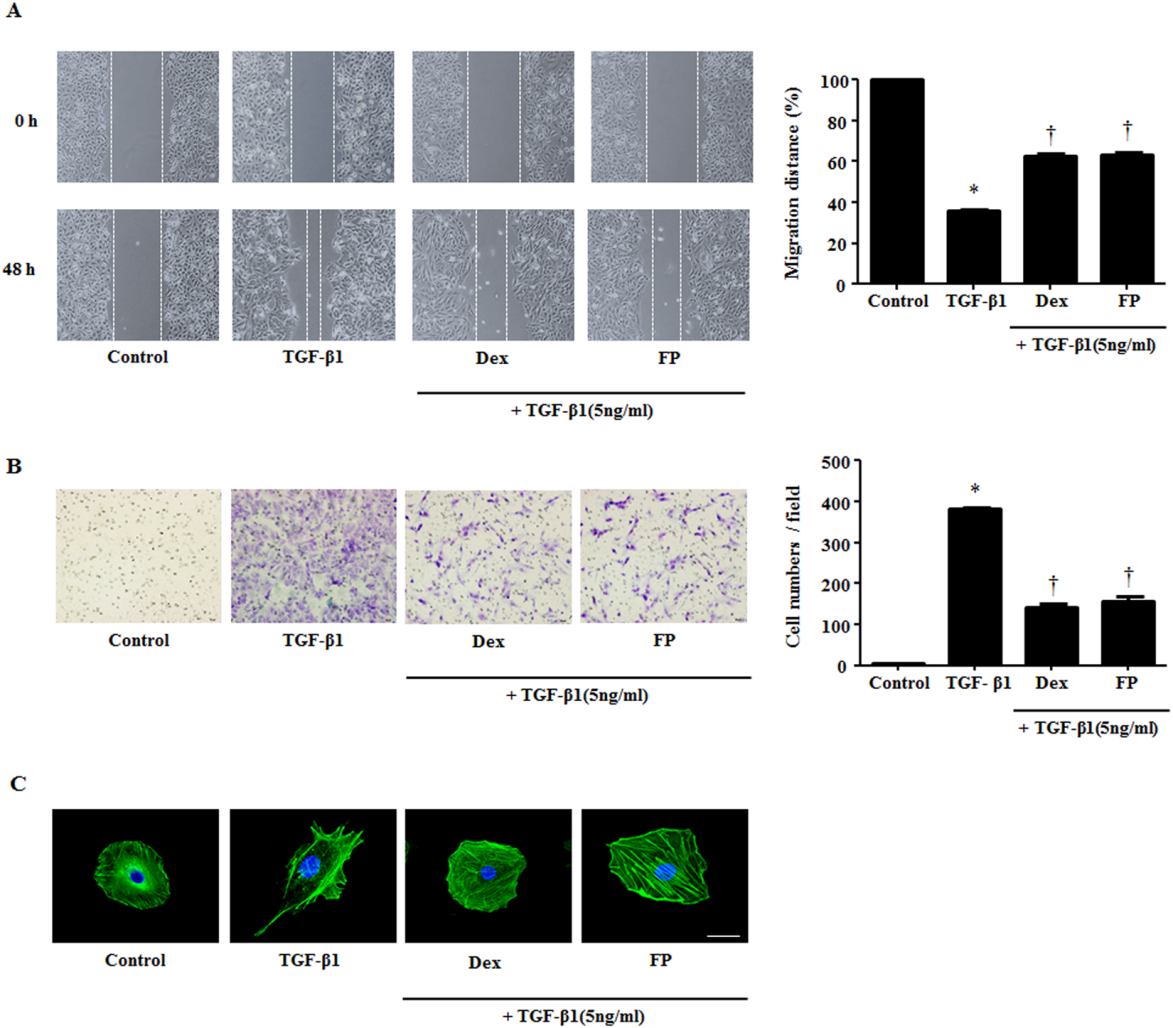
Glucocorticoids mediate migratory and invasive properties stimulated by TGF-β1 in A549 cells. Cells were pretreated with or without dexamethasone (Dex, 2.5 μM) and fluticasone propionate (FP, 2.5 μM) for 1 hour and then stimulated with TGF-β1 (5 ng/ml). (**A**) Wound scratch and (**B**) Transwell migration assays established migration and invasion by A549 cells. Representative images are shown. In the wound scratching assays, the graphic representation is the percent of migrated cells counted within the areas of healing covering the lines present at baseline. In the Transwell migration assays, invasive cells were counted in five high-power fields (HPFs) for the average cell number migrated per HPF. (**C**) Visualization of F-actin used phalloidin with DAPI staining. Immunofluorescence was determined by confocal laser scanning microscope. Scale bar = 20 μm. Data are presented as means ± SEM. Results were from at least three independent experiments. *p < 0.05 vs. control; ^†^p < 0.05 vs. TGF-β1.

In EMT, epithelial cells change into mesenchymal cells and acquire migratory and invasive properties^11, 12^. Therefore, to explore whether TGF-β1 and GCs influence the motile activity of A549 cells, we performed wound scratch and Transwell migration assays. Migration distance after TGF-β1 treatment was reduced up to 64.2%, and the number of migrated cells increased up to 400 compared with the control group. Pretreatment with GCs reversed these effects, inhibiting the cells’ motility capacity (Fig. 2A and B). To support this finding, we confirmed the expression of filamentous actin in A549 cells by staining with phalloidin and 4′,6-diamidino-2-phenylindole. The TGF-β1-treated A549 cells spread out and flattened in shape. Treatment with GCs had the opposite effect of TGF-β1, in line with the migration data (Fig. 2C). Our results revealed that treatment with GCs could be an effective strategy for addressing TGF-β1-mediated EMT in airway epithelial cells, suggesting that GCs regulate cellular motility through alteration of EMT-associated proteins.

### Glucocorticoids prevented EMT by inhibiting the TGF-β1-induced mitogen-activated protein kinase (MAPK) pathway

**Figure 3:**
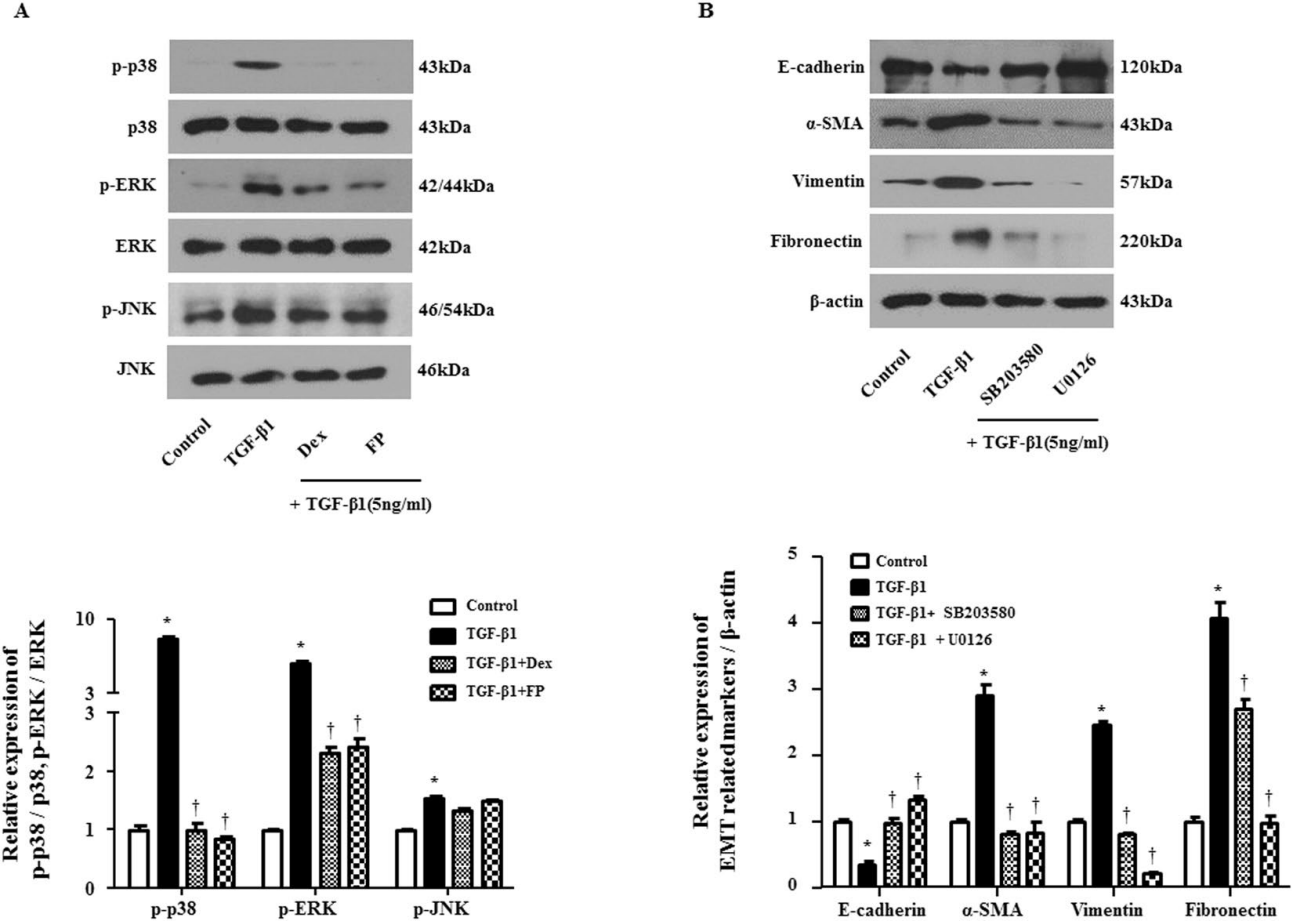
Glucocorticoids mediate MAPK signaling pathway by TGF-β1 in A549 cells. Cells were pretreated with or without dexamethasone (Dex, 2.5 μM) and fluticasone propionate (FP, 2.5 μM) or the MAPK pathway inhibitors of p38 (SB203580, 10 μM) or ERK (U0126, 10 μM) and then stimulated with TGF-β1 (5 ng/ml) for 30 minutes. (**A**) Phosphorylation of MAPK signaling molecules: p-p38, p-ERK and total MAPK signaling molecules were determined by Western blot. Data were normalized to total p38 or ERK expression. (**B**) Protein levels of EMT-related markers were determined by Western blot. Data were normalized to β-actin. Data are presented as mean ± SEM. Results were from at least three independent experiments. *p < 0.05 vs. control; ^†^p < 0.05 vs. TGF-β1.

Activation of ERK by TGF-β1 induces the loss of cell-cell adhesion and polarity and induces the mobility of cells and phosphorylation of the p38/JNK pathway involved in cell morphology changes and cytoskeleton reorganization under EMT^20^. Thus, using western blotting, we investigated whether GCs activated the MAPK pathway to control TGF-β1-induced EMT. In agreement with previous studies^21^, cells treated with TGF-β1 exhibited enhanced phosphorylation of p38 and ERK. In contrast, GCs significantly down-regulated the phosphorylation of ERK and p38 (P < 0.05, Fig. 3A) compared with that of TGF-treated cells. GCs did not affect the activation of JNK after TGF-β1 treatment in A549 cells. To ensure that signaling was associated with EMT, the p38 inhibitor SB203580 and ERK inhibitor U0126 were used for pretreatment for 1 hour after stimulation with TGF-β1 for 72 hours. E-cadherin was reduced by TGF-β1 and recovered with MAPK inhibitors. Mesenchymal markers α-SMA, vimentin and fibronectin were induced by TGF-β1 and reduced by MAPK inhibitors (Fig. 3B). These results showed that the phosphorylation of p38 and ERK and the activation of EMT markers induced by TGF-β1 decreased in airway epithelial cells after GC treatment in a manner similar to that observed after treatment with SB203580 and U0126 as p38- and ERK-specific inhibitors, respectively, suggesting that the p38 and ERK signaling pathways, not JNK, could be responsible for GC-mediated EMT.

### Glucocorticoids prevented TGF-β1-induced Snail and Slug expression, and regulation of Snail and Slug ameliorated TGF-β1-induced EMT in A549 cells

**Figure 4:**
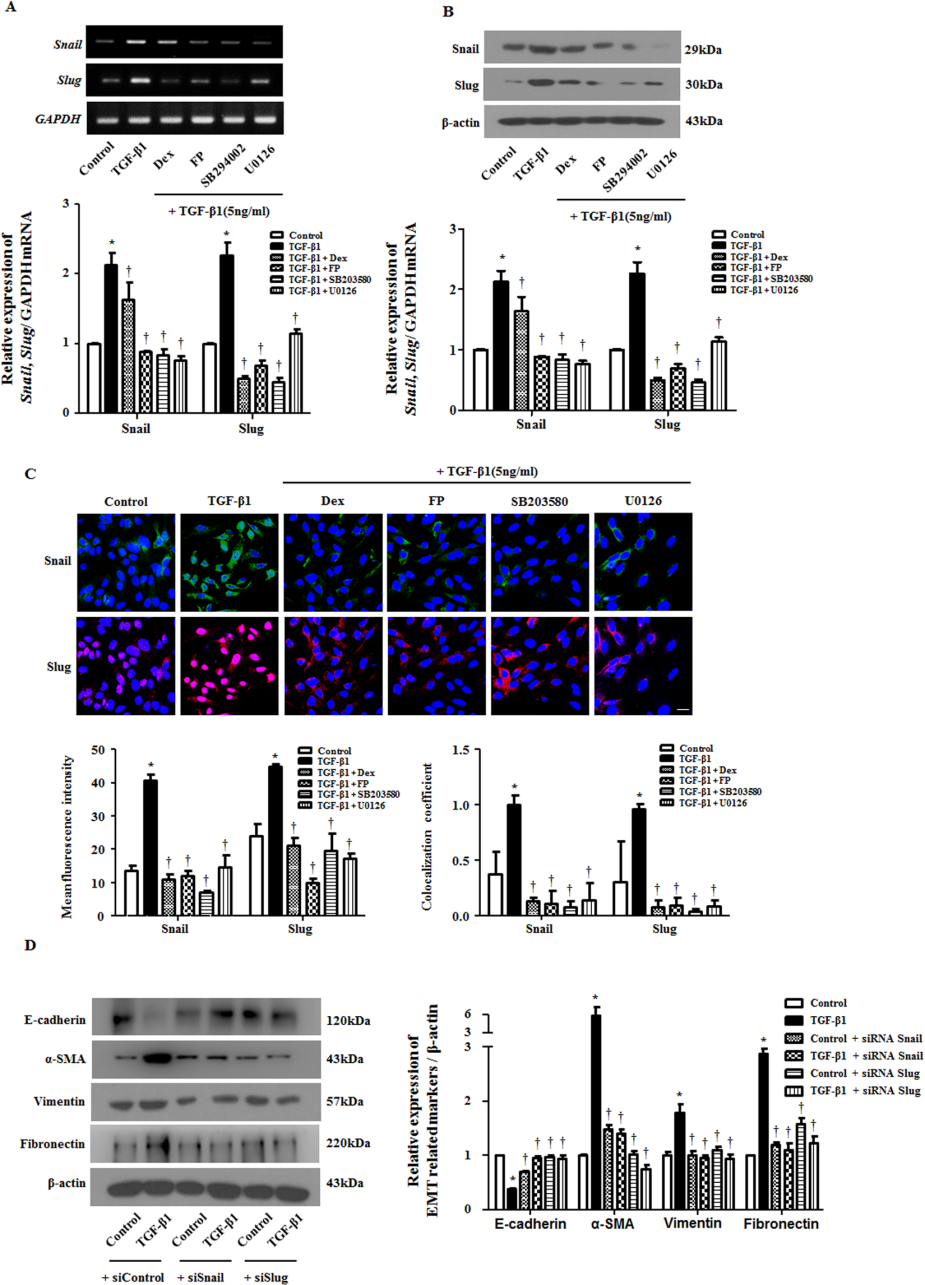
Glucocorticoids prevent Snail and Slug expression by TGF-β1 in A549 cells. Cells were pretreated with or without dexamethasone (Dex, 2.5 μM) and fluticasone propionate (FP, 2.5 μM), MAPK pathway inhibitors of p38 (SB203580, 10 μM) or ERK (U0126, 10 μM) and then stimulated with TGF-β1 (5 ng/ml). The mRNA and protein levels of Snail/Slug were determined by (**A**) semiquantitative RT - PCR and (**B**) Western blot, which were normalized to GAPDH and β-actin, respectively. (**C**) To confirm the expression and translocation of Snail and Slug, immunofluorescence was performed, and images were acquired by confocal laser scanning microscope. Representative fluorescein immunocytochemical staining is shown with Snail (green), Slug (red) and nuclear DAPI (blue). Scale bar = 20 μm. (**D**) Specific Snail and Slug siRNAs (100 nM) were transiently transfected before treatment with or without TGF-β1 (5 ng/ml) for 72 hours. EMT-related markers were determined by Western blots. Data are presented as mean ± SEM. Results were from at least three independent experiments. *p < 0.05 vs. control; ^†^p < 0.05 vs. TGF-β1.

Snail and Slug are zinc-finger transcriptional repressors that regulate E-cadherin expression by binding E-boxes. Through this process, they induce EMT^15, 16^. To examine whether TGF-β1 promotes Snail and Slug expression and whether GCs abolish the EMT process via Snail and Slug, reverse transcription polymerase chain reaction (RT-PCR) and Western blots were performed. In the results of these, Snail and Slug transcripts were up-regulated by TGF-β1 and suppressed by GCs, SB294002 (p38 specific inhibitor) and U0126 (ERK-specific inhibitor) (Fig. 4A and B). GCs, SB294002 and U0126 prevented the overexpression and translocation of Snail and Slug from the cytoplasm to the nucleus, blocking the action of TGF-β1 (Fig. 4C). To determine whether Snail- and Slug-controlled EMT markers were down-regulated in A549 cells, we detected EMT marker proteins after Snail- and Slug-specific small interfering RNA (siRNA) transfection. The Knockdown of Snail and Slug with siRNA was confirmed by RT - PCR. Western blots showed that the depletion of Snail and Slug ameliorated TGF-β1-induced variation in EMT-related markers in A549 cells (Fig. 4D). These data suggested that Snail and Slug were critical for modulating GC-mediated EMT after TGF-β1 stimulus in airway epithelial cells.

### Glucocorticoids suppressed TGF-β1-induced EMT in primary nasal epithelial cells

**Figure 5:**
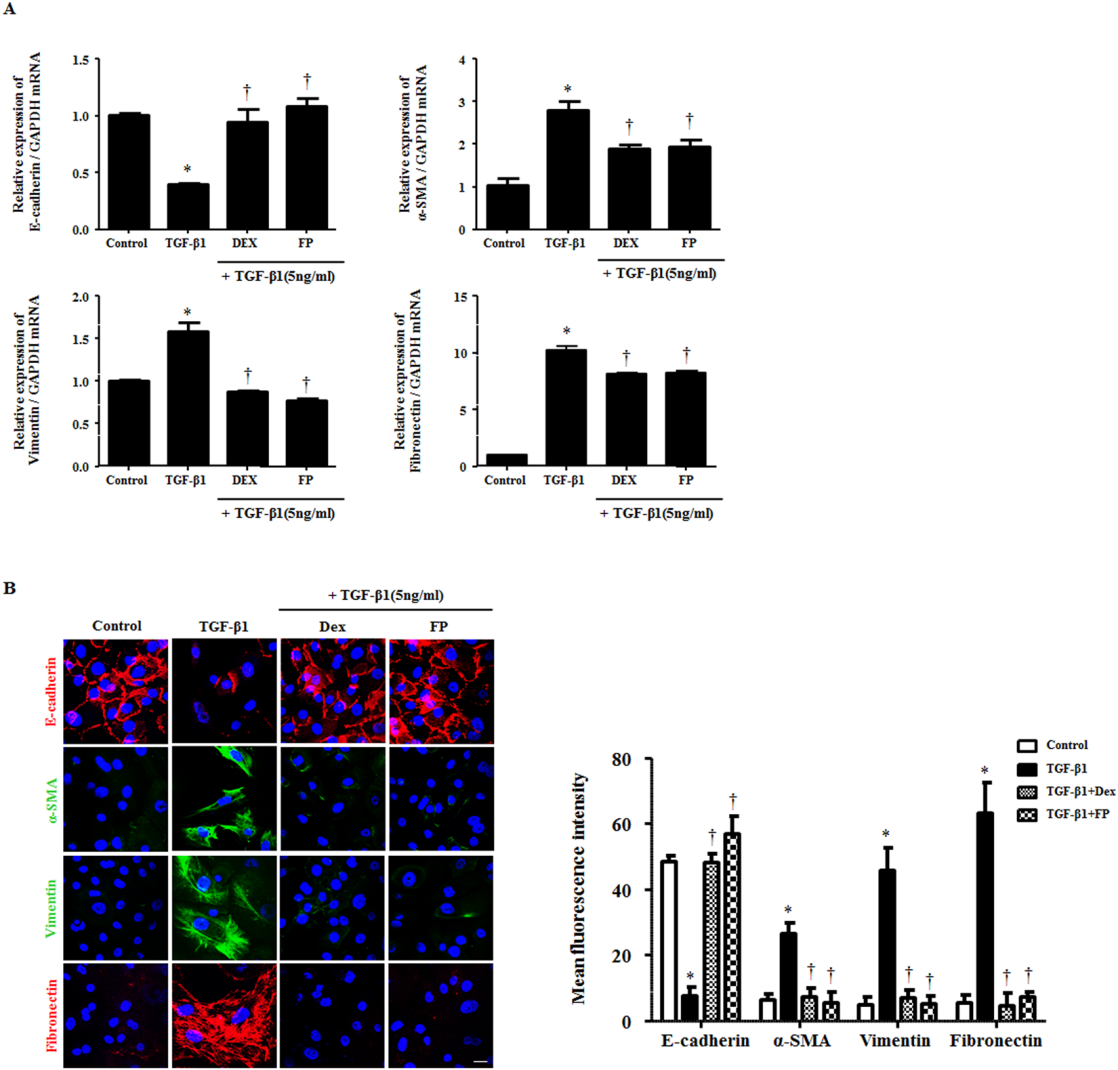
Glucocorticoids modulate EMT-related markers that were stimulated by TGF-β1 in human primary nasal epithelial cells. Cells were pretreated with or without dexamethasone (Dex, 2.5 μM) and fluticasone propionate (FP, 2.5 μM) and then stimulated with TGF-β1 (5 ng/ml). (**A**) The mRNA for EMT-related markers was determined by real-time PCR. Data were normalized to GAPDH. (**B**) The protein levels and localization of EMT-related markers were observed by immunofluorescence. Images were acquired by confocal laser scanning microscope. Representative fluorescein immunocytochemical staining is depicted with E-cadherin (red), α-SMA (green), vimentin (green), fibronectin (red) and nuclear DAPI (blue). Scale bar = 20 μm. Data are presented as mean ± SEM. Three primary cell lines from different donors were used. Experiments were performed in at least triplicate and repeated at least three times. *p < 0.05 vs. control; ^†^p < 0.05 vs. TGF-β1.

To test the effect of GCs on TGF-β1-induced EMT, we further investigated mRNA and protein levels for EMT in human primary nasal epithelial cells (hPNECs). In agreement with the data on A549 cells, real-time PCR assays showed that TGF-β1 treatment significantly initiated the transcription of EMT markers, while GCs significantly blocked the activation of EMT-related proteins in hPNECs (Fig. 5A). Upon immunofluorescent evaluation, the results were consistent with data using A549 cells in immunofluorescence (Fig. 5B). These results indicated that GCs governed and regulated the tissue remodeling involved in EMT in the TGF-β1-mediated nasal epithelium.

### Glucocorticoids inhibited TGF-β1-induced EMT in *ex vivo* organ culture of the nasal inferior turbinate

**Figure 6:**
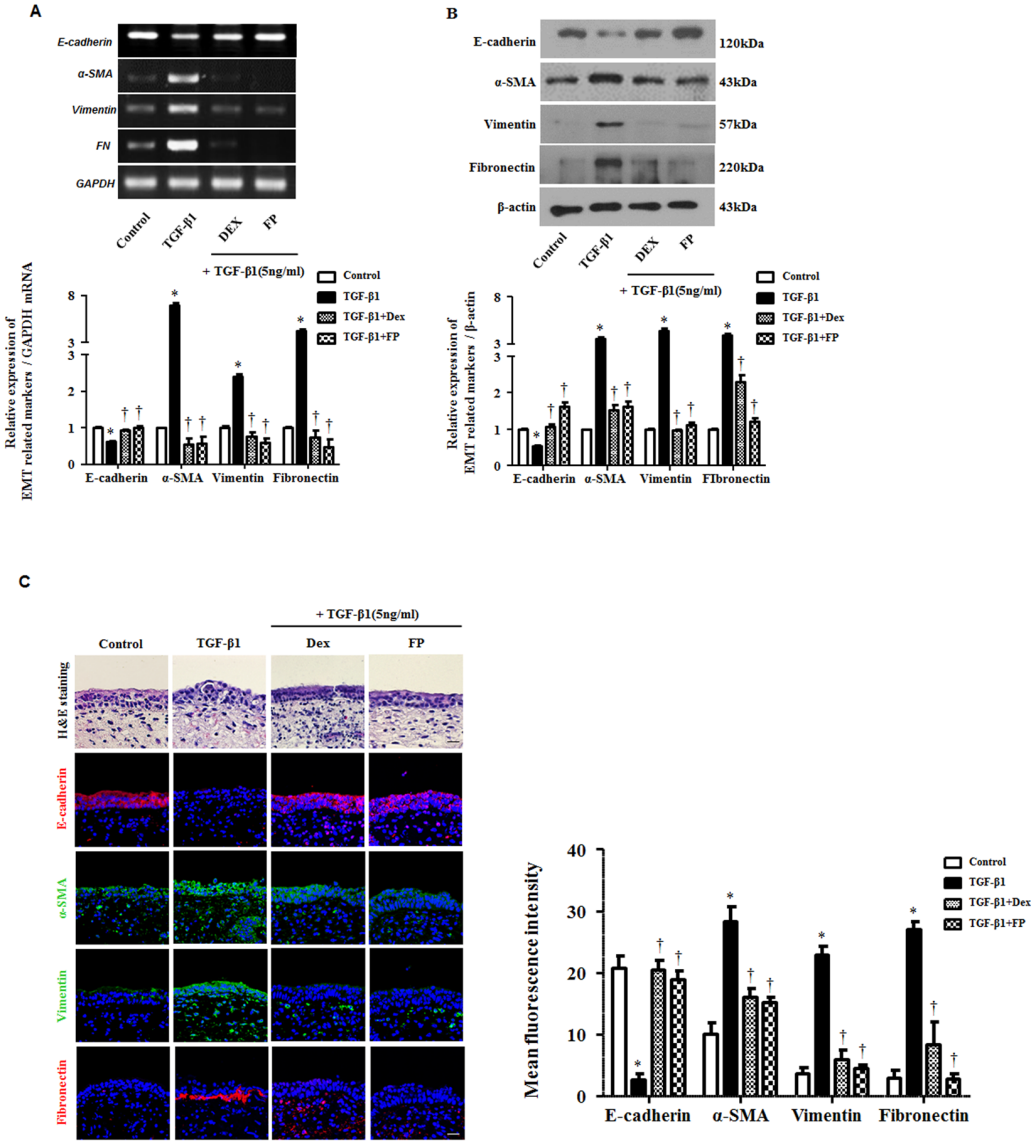
Glucocorticoids reduce TGF-β1-induced EMT in *ex vivo* organ cultures of the nasal inferior turbinate. Nasal inferior turbinate tissues were cultured and pretreated with or without dexamethasone (Dex, 2.5 μM) and fluticasone propionate (FP, 2.5 μM) and then stimulated with TGF-β1 (10 ng/ml). The mRNA for EMT-related markers was determined by (**A**) semiquantitative RT-PCR and (**B**) western blot. Data were normalized to GAPDH and β-actin expression. (**C**) Representative hematoxylin and eosin and fluorescein immunohistochemical staining for E-cadherin (red), α-SMA (green), vimentin (green), fibronectin (red) and nuclear DAPI (blue). Scale bar = 50 μm. Data are presented as mean ± SEM. *Ex vivo* organ cultures from different donors were used. Results were from at least three independent experiments. *p < 0.05 vs. control; ^†^p < 0.05 vs. TGF-β1.

To assess whether GCs affected alterations in the mRNA and proteins mediated by EMT in nasal inferior turbinate tissue, we cultured *ex vivo* nasal inferior turbinate organs following an air-liquid interface culture method^10^. Expression of α-SMA, vimentin, and fibronectin increased with TGF-β1 and was suppressed by GCs, while E-cadherin was expressed in the opposite pattern (Fig. 6A and B) in mRNA and protein levels. Furthermore, the immunohistochemical staining showed similar patterns. In fibronectin, deposition was observed in the lower part of the epithelial layer in TGF-β1-treated tissue. Pretreatment with GCs prevented alterations in EMT markers by TGF-β1 (Fig. 6C). These results suggested that GCs could regulate EMT-associated protein expression by blocking the MAPK and Snail/Slug signaling pathways in the nasal epithelium in the presence of TGF-β1.

## Discussion

In this study, we examined the effects and underlying mechanisms of GC action on TGF-β-induced EMT in airway epithelium. The loss of membranous E-cadherin and gain of α-SMA, vimentin and fibronectin are hallmarks of airway epithelial cells undergoing EMT, which results in morphological changes^13, 14^. Treatment with TGF-β1 allows epithelial cells to change from cuboidal to elongated spindle-like shapes and alters their expression of epithelial and mesenchymal markers, while augmenting cellular motility^22^. Our data showed that pretreatment with GCs ameliorated EMT-related alteration and inhibited migration by down-regulating MAPK and Snail/Slug expression in TGF-β1-induced A549 cells. These results were consistent with our data using hPNECs and *ex vivo* organ cultures of the inferior turbinate.

Although the etiology remains unclear, CRS is a chronic inflammatory disease in the sinus and is associated with numerous and extensive inflammatory processes involving both structural and infiltrating inflammatory cells that produce various cytokines and mediators^23, 24^. This cytokine is postulated to be involved in airway remodeling^25, 26^. TGF-β1, a well-known stimulator of EMT, induces the transition of epithelial cells into myofibroblasts, contributing to tissue remodeling and the pathogenesis of CRSwNP^10, 27^. Early-stage nasal polyps exhibit increased TGF-β1 levels and more epithelial loss and induced myofibroblast differentiation with activated α-SMA and vimentin. This EMT phenomenon is mainly seen in the stalks of early stage nasal polyps rather than the nasal polyp bodies. Therefore, TGF-β1 expressed in the stalks of nasal polyps will play an important role in the formation of the nasal polyps by inducing EMT^28^.

EMT is a process that transdifferentiates epithelial cells into mesenchymal cells such as fibroblasts and myofibroblasts, and as such, it is important in stem cell behavior, embryonic development and wound healing. During TGF-β1-induced EMT, epithelial cells lose cell-cell junctions, inducing their separation from encompassing cells, and acquire mesenchymal-like characteristics. These changes allow the cells to move from their original sites and are accompanied by altered expression of EMT-related markers^22, 29^. In agreement with previous reports on the TGF-β1 response, our data demonstrated that E-cadherin as an epithelial marker was decreased, while α-SMA, vimentin, and fibronectin as mesenchymal markers were increased.

Therapy with GCs such as dexamethasone and fluticasone propionate, which are extensively used as anti-inflammatory drugs, treat a variety of human inflammatory diseases^30, 31^. In clinical rhinology, GC treatment is considered the first and limited efficient treatment of patients with CRSwNP. GCs were reported to be highly effective at alleviating nasal symptoms and nasal airflow, improving life quality and shrinking nasal polyps^32^. Dexamethasone also reduced the transcription of EMT-related genes such as those for vimentin and fibronectin in hepatocytes^18^ and blocked TGF-β1-induced EMT and cell migration in lung epithelial cells^33^. Chanez *et al*. reported that dexamethasone strengthened epithelial cell-cell adhesion by inducing E-cadherin expression in nasal epithelium^34^. The effects of inhaled fluticasone propionate have also been reported to have anti-epithelial activation and anti-EMT effects in chronic obstructive pulmonary disease^19^. However, the effects of GCs on EMT in the pathogenesis of CRS remain unknown. We thus hypothesized that GCs caused anti-EMT effects in TGF-β1-treated airway epithelial cells and in *ex vivo* organ cultures of the nasal inferior turbinate. However, the effects of dexamethasone on EMT is controversial and dependent on concentration. Dexamethasone treatment with only 100 nM regulated *α-*SMA and collagen type I expression induced by TGF-β1 in BEAS-2B and primary normal human bronchial epithelial cells^35^. In contrast, 10 μM of dexamethasone completely suppressed TGF-β1-induced EMT in human peritoneal mesothelial cells^36^. Therefore, we conducted experiments at various concentrations of GCs to determine the appropriate concentrations needed to inhibit TGF-β1-induced EMT and selected the most optimal concentrations.

Although TGF-β/Smad signaling is regarded as the main pathway in EMT, TGF-β activates non-Smad signaling pathways as well, including MAPK pathways, Rho-like GTPase signaling pathways and phosphatidylinositol-3-kinase/AKT pathways^20^. Several studies have demonstrated that the activation of ERK and p38 is mediated by TGF-β during EMT in lung epithelial cells^29, 37^. In particular, ERK activation is necessary for changes in morphological features and the disassembly of cell adherent junctions as well as the acquisition of motile and invasive properties after TGF-β1 treatment^38^. Previous reports have indicated that the effect of TGF-β1 on ERK and p38 phosphorylation is decreased by GCs. However, the effect of TGF-β1 on Smad2/3 phosphorylation is not inhibited by GCs. This result suggests that the effect of dexamethasone is achieved by blocking EMT through the non-Smad pathway in human peritoneal mesothelial cells^36^. In our study with A549 cells, ERK and p38 phosphorylation was seen after treatment with TGF-β1, whereas GCs down-regulated the TGF-β1-promoted activation of p38 and ERK. The effects of dexamethasone on the Smad signaling pathway remain controversial^18, 36^. Given that the effect of GCs on EMT via Smad or non-Smad is a matter of debate, the different effects of GCs on the Smad and non-Smad signaling pathways may be cell type-specific and dependent on the upper airway environment. These findings indicate that EMT processes involve numerous complex factors in Smad and non-Smad signaling pathways.

Recent evidence suggests that EMT is regulated by a number of transcription factors, such as Snail and Slug. The Ectopic expression of Snail and Slug down-regulates E-cadherin expression and up-regulates vimentin and fibronectin expression, leading to a full EMT phenotype^15, 16, 39^. As observed in several studies, TGF-β1 has key activity in the EMT program via both Smad and non-Smad pathways, and induces Snail and Slug^40^. Our study demonstrated that Snail and Slug transcription factors were down-regulated, and the translocation of the factors from the cytoplasm into the nucleus was inhibited, under the response to GCs and p38/ERK-specific inhibitors during TGF-β1-induced EMT. These results suggested GCs contributed to anti-tissue remodeling via EMT. Moreover, we showed that the siRNA-mediated knockdown of Snail and Slug counteracted EMT-related protein expression. This result suggested that the overexpression of Snail and Slug in airway epithelial cells interrupted EMT action by TGF-β1 through Snail and Slug signaling pathways.

Our *ex vivo* organ culture data also showed that the effects of GCs in TGF-β1-induced EMT were closely related to the regulation of EMT-related transcripts. Since an *in vivo* model system of nasal polyps has not been established, a nasal *ex vivo* organ culture model was used to study the etiology of nasal polyps. Our *ex vivo* organ culture system showed that GCs significantly modulated TGF-β1-induced EMT at both the mRNA and protein levels. We also observed positivity for epithelial markers and negative staining for mesenchymal markers using confocal microscopy of *ex vivo* organ cultures of the inferior turbinate.

**Figure 7:**
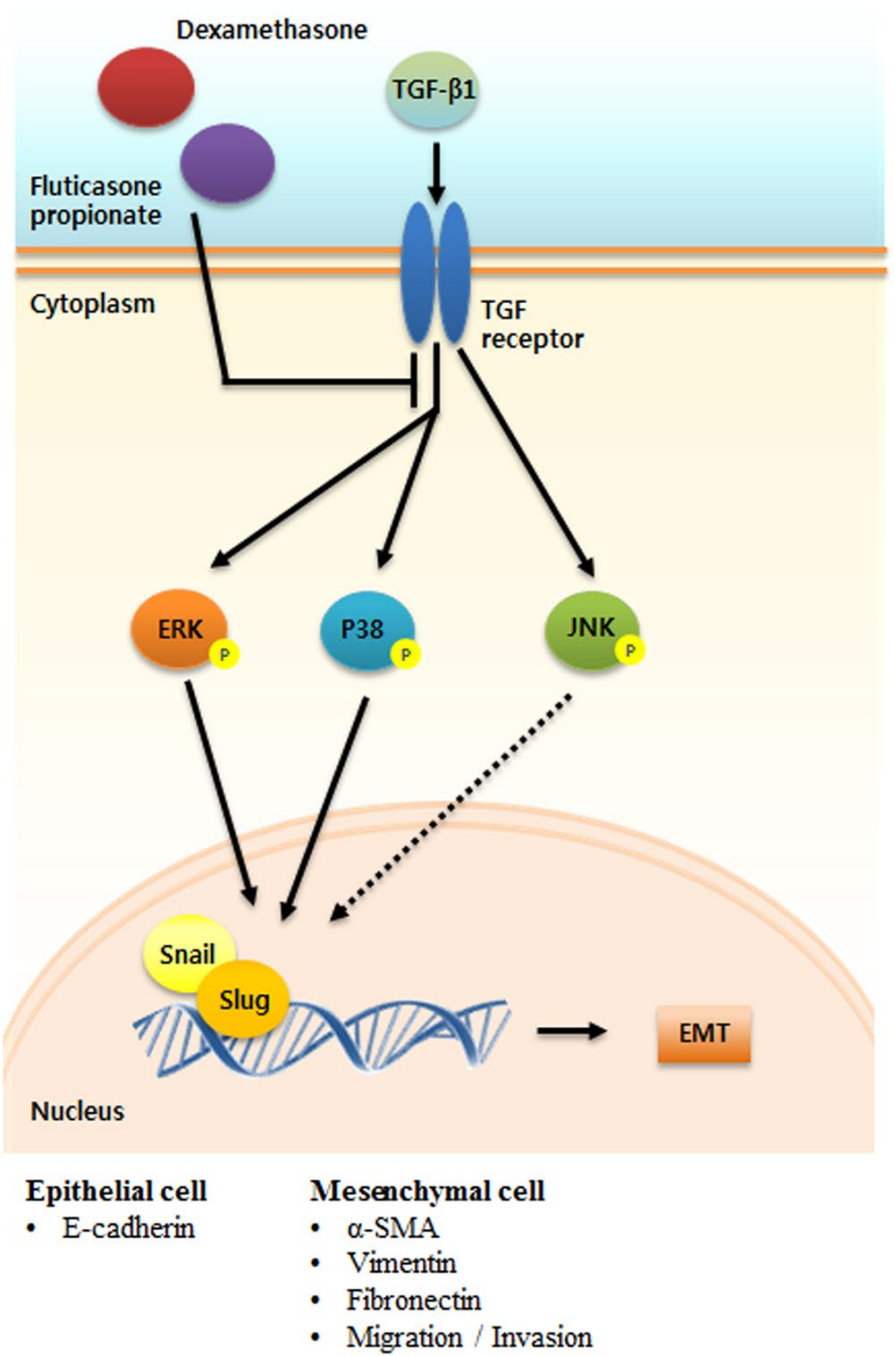
Hypothetical schema of the role of glucocorticoids in TGF-β1-induced EMT in airway epithelial cells and nasal *ex vivo* organ culture. GCs modulate EMT-related proteins; E-cadherin, α-SMA, vimentin and fibronectin by inhibiting the p38/ERK and Snail/Slug signaling pathways, resulting in restrained migration and invasion in the airway epithelium under EMT mediated by TGF-β1.

Based on our evidence, we propose a model that supports the anti-tissue remodeling activity of GCs in the airway epithelium. This activity is exerted via EMT processes by reversing the expression of E-cadherin and inhibiting cellular motility through the MAPK and Snail or Slug signaling pathways (Fig. 7). We described a mechanism by which dexamethasone and fluticasone propionate exerted anti-EMT activity under TGF-β1-stimulated EMT processes such as fibroblast-like morphological changes and alterations in EMT-related protein expression. This effect resulted in enhanced cellular motility efficacy in airway epithelial cells and *ex vivo* organ cultures of the nasal inferior turbinate. The Model shows that GCs function as useful therapeutic agents by recovering E-cadherin protein expression, suppressing the expression of *α-*SMA, vimentin and fibronectin and subsequently inhibiting cellular migration, ultimately reducing tissue remodeling. GCs may have anti-EMT activities such as regulating EMT-related protein expression and the function of cellular motility in airway epithelial cells. GCs act on tissue remodeling under the TGF-β1 pool through the MAPK and Snail/Slug signaling pathways, which could potentially contribute to the prevention and treatment of CRSwNP.

## Methods

### Inferior turbinate tissues

For hPNECs and *ex vivo* organ cultures, the inferior turbinate mucosa was obtained from eight patients (five males and three females; mean age 43.1 years) during endoscopic sinus surgeries for or rhinoplasty at the Department of Otorhinolaryngology, Korea University Medical Center, Korea. All tissues were from patients with no signs of inflammation, allergies, asthma or aspirin sensitivity. No patients had taken oral steroids, non-steroidal anti-inflammatory drugs, antihistamines or antibiotics for at least 2 months. Written informed consent was provided according to the Declaration of Helsinki. This study was approved by the Korea University Medical Center Institutional Review Board, which also authorized the research (KUGGR-12041-001), and it was carried out in accordance with the guidelines of the Human Ethics Committee of the Korea University Guro Hospital.

### Cell culture

Human airway epithelial cell line A549 (ATCC CCL-185; Rockville, MD) was from the American Type Culture Collection. Cells were cultured in RPMI1640 medium supplemented with 10% fetal bovine serum (FBS), 1% 10,000 units/ml penicillin and 1% 10,000 μg/ml streptomycin (Invitrogen, Carlsbad, CA) in an incubator at 37 °C and 5% CO_2_.

To isolate hPNECs from the nasal inferior turbinate, tissues were washed with 1× phosphate-buffered saline (PBS) and immersed in dispase (Stem Cell Technologies, Vancouver, Canada) for 4 hours before the hPNECs were separated with a 100-nm strainer. hPNECs were cultured in airway epithelial cell growth medium with Supplement-Mix (Promocell, Heidelberg, Germany). The purity of the hPNECs was confirmed through positive staining for cytokeratin 18 by immunocytochemistry (data not shown).

### Western blotting

A549 cells and hPNECs were seeded into 60-mm culture dishes with 8 × 10^5^ cells/ml. Cells were lysed in radioimmunoprecipitation assay buffer (Sigma, St. Louis, MO, USA) with protease inhibitors and phosphatase inhibitors (Sigma). Proteins were separated by 10% sodium dodecyl sulfate polyacrylamide gel electrophoresis and transferred onto polyvinyl difluoride membranes (Millipore Inc., Billerica, MA, USA). Membranes were blocked with 5% skim milk. The blots were incubated with primary antibodies against E-cadherin, vimentin, fibronectin, phospho-p38, total-p38 Snail, β-actin (Santa Cruz Biotechnology, Santa Cruz, CA, USA), α-SMA, phospho-JNK, total-JNK (Millipore Inc.) phospho-ERK, total-ERK and Slug (Cell Signaling Technology, Danvers, MA, USA). The blots were visualized with horseradish peroxidase-conjugated secondary antibodies and enhanced chemiluminescent system (Pierce, Rockford, IL, USA).

### Immunofluorescence of airway epithelial cells and inferior turbinate tissues

Immunofluorescence assays were carried out as previously described^10^. Briefly, A549 cells, hPNECs and *ex vivo* organ cultures of the inferior turbinate were stained with primary antibodies, then incubated with anti-mouse Alexa 488 and anti-rabbit Alexa 555 secondary antibodies and finally counterstained with 4′-6-diamidino-2-phenylindole (DAPI; Sigma). To assess changes in the actin cytoskeleton, phalloidin (F-actin) staining was conducted with phalloidin antibody (Thermo Fisher Scientific, Waltham, MA, USA). Image acquisition and processing were performed using a confocal laser scanning microscope LSM700 (Zeiss, Oberkochen, Germany). Expression of EMT-markers and colocalization of Snail/Slug and DAPI were determined by mean fluorescence intensity and Manders’ overlap coefficient analysis using image software^41, 42^.

### Hematoxylin and eosin staining

All tissues were fixed in 4% paraformaldehyde for 24 hours and washed with PBS. Tissues were passed through a dehydration process and embedded in paraffin. Paraffin blocks were sectioned at a 4-μm thickness for hematoxylin and eosin staining.

### Semiquantitative and quantitative RT-PCR

To evaluate mRNA levels in primary nasal epithelial cells, semiquantitative and quantitative RT - PCR assays were performed. Total RNA was extracted by a TRIzol reagent (Invitrogen). Reverse transcription was performed using Moloney murine leukemia virus reverse transcriptase (Invitrogen) according to the manufacturer’s instructions. Semiquantitative RT - PCR was by Molecular Imager ChemiDoc XRS+ (Bio-Rad, Hercules, CA, USA). Quantitative RT - PCR was performed with Quantstudio3 (Applied Biosystems, Foster City, CA, USA) using Power SYBR Green PCR Master Mix (Applied Biosystems). Relative gene expression was calculated by evaluating quantitative RT - PCR data using the 2 (2DDCt) method. Experiments were repeated at least three times, and glyceraldehyde 3 -phosphate dehydrogenase (GAPDH) was the internal control. Forward and reverse primers for PCR are shown in Table 1.

**Table 1:** Sequence of primers for semiquantitative and quantitative RT-PCR.

Gene Name		Sequences (semiquantitative RT-PCR)	Sequences (quantitative RT-PCR)
E-cadherin	Forward	5′ -TGC TCT TGC TGT TTC TTC GG-3′	5′-TGC TCT TGC TGT TTC TTC GG-3′
	Reverse	5′-TGC CCC ATT CGT TCA AGT AG-3′	5′-TGC CCC ATT CGT TCA AGT AG-3′
α-SMA	Forward	5′-GGT GCT GTC TCT CTA GCC TCT GGA-3′	5′-GGC TCT GGG CTC TGG GCT TCA TC-3′
	Reverse	5′-CCC ATC AGG CAA CTC GAT ACT CTT C-3′	5′-CTC TTG CTC TGG GCT TCA TC-3′
Vimentin	Forward	5′-GAA GAG AAC TTT GCC GTT GAA G-3′	5′-GAA GAG AAC TTT GCC GTT GAA G-3′
	Reverse	5′-GAG AAA TCC TGC TCT CCT CG-3′	5′-GAG AAA TCC TGC TCT CCT CG-3′
Fibronectin	Forward	5′-GGA TGC TCC TGC TGT CAC-3′	5′-CTT TGG TGC AGC ACA ACT TC-3′
	Reverse	5′-CTG TTT GAT CTG GAC CTG CAG-3′	5′-CCT CCT CGA GTC TGA ACC AA-3′
GAPDH	Forward	5′-GTG GAT ATT GTT GCC ATC AAT GAC C-3′	5′-GTG GAT ATT GTT GCC ATC AAT GAC C-3′
	Reverse	5′-GCC CCA GCC TTC TTC ATG GTG GT-3′	5′-GCC CCA GCC TTC TTC ATG GTG GT-3′
Snail	Forward	5′-TCT AGG CCC TGG CTG CTA CAA-3′	
	Reverse	5′-GCC TGG CAC TGG TAC TTC AC-3′	
Slug	Forward	5′-ATG CAT ATT CGG ACC CAC C-3′	
	Reverse	5′-AGA TTT GAC CTG TCT GCA GCT C-3′	

### Wound scratch and Transwell migration assays

Wound scratch and Transwell migration assays were conducted as previously described^10^. A549 cells were pretreated with dexamethasone (2.5 μM) or fluticasone propionate (2.5 μM) for 1 hour and then stimulated with TGF-β1 (5 ng/ml). For the wound scratch migration assays, cells were photographed with a digital camera (Olympus BX51; Olympus, Tokyo, Japan) 48 hours after the scratch. Wound closure of the cells that crossed into the scratch area as compared to zero time was calculated by ImageJ software (National Institutes of Health, Bethesda, MD, USA). Graphs represent the percentage wound closure. For the Transwell migration assays, A549 cells from the migrated side were stained with Diff-Quik stain (Sysmex, Kobe, Japan). Images were acquired under a microscope at 200× magnification (Olympus BX51; Olympus), and stained cells were counted in five high-power fields (HPFs) to determine the average cell number migrated per HPF.

### Transient knockdown of Snail or Slug with siRNA

All siRNAs (Santa Cruz Biotechnology) were transfected into A549 cells using Lipofectamine 2000 (Invitrogen). After 24 and 48 hours, total RNA was isolated from cells as for semiquantitative and quantitative RT-PCR, and knockdown of the Snail or Slug gene was confirmed by quantitative RT - PCR.

### *Ex vivo* organ culture

*Ex vivo* organ culture of the nasal inferior turbinate was performed as described previous study^10^. Nasal inferior turbinate tissues were cut into 2- to 3-mm^3^ pieces, washed three times with PBS and cultured in Dulbecco’s modified Eagle’s medium supplemented with 2% FBS (Invitrogen), 1% 10,000 units/ml penicillin and 1% 10,000 μg/ml streptomycin (Invitrogen). Nasal inferior turbinate tissues were pretreated with or without dexamethasone (5 µM) or fluticasone propionate (5 µM) and stimulated with TGF-β1 (10 ng/ml) for 72 hours.

### Statistical analysis

Results were obtained from at least three independent experiments. Statistical significance of differences between control and experimental data was analyzed using the unpaired t-test or one-way analysis of variance followed by Tukey’s test (GraphPad Prism, version 5, GraphPad Software, San Diego, CA). Significance was established at the 95% confidence level. P-values less than 0.05 were accepted as statistically significant.

## References

[1] Fokkens WJ (2012). EPOS 2012: European position paper on rhinosinusitis and nasal polyps 2012. A summary for otorhinolaryngologists. Rhinology.

[2] Eggesbo HB (2012). Imaging of sinonasal tumours. Cancer imaging: the official publication of the International Cancer Imaging Society.

[3] Newton JR, Ah-See KW (2008). A review of nasal polyposis. Therapeutics and clinical risk management.

[4] Esmatinia F (2014). Recurrent sinonasal polyposis after the endoscopic sinus surgery. Reviews in Clinical Medicine.

[5] Van Bruaene N, Bachert C (2011). Tissue remodeling in chronic rhinosinusitis. Current opinion in allergy and clinical immunology.

[6] Nawshad A, Lagamba D, Polad A, Hay ED (2005). Transforming growth factor-beta signaling during epithelial-mesenchymal transformation: implications for embryogenesis and tumor metastasis. Cells, tissues, organs.

[7] Shin HW (2012). Hypoxia-inducible factor 1 mediates nasal polypogenesis by inducing epithelial-to-mesenchymal transition. American journal of respiratory and critical care medicine.

[8] Niessen CM (2007). Tight junctions/adherens junctions: basic structure and function. The Journal of investigative dermatology.

[9] Hupin C (2014). Features of mesenchymal transition in the airway epithelium from chronic rhinosinusitis. Allergy.

[10] Park IH, Kang JH, Shin JM, Lee HM (2016). Trichostatin A Inhibits Epithelial Mesenchymal Transition Induced by TGF-beta1 in Airway Epithelium. PloS one.

[11] Kalluri R, Neilson EG (2003). Epithelial-mesenchymal transition and its implications for fibrosis. The Journal of clinical investigation.

[12] Lamouille S, Xu J, Derynck R (2014). Molecular mechanisms of epithelial-mesenchymal transition. Nature reviews. Molecular cell biology.

[13] Kalluri R, Weinberg RA (2009). The basics of epithelial-mesenchymal transition. The Journal of clinical investigation.

[14] Thiery JP (2002). Epithelial-mesenchymal transitions in tumour progression. Nature reviews. Cancer.

[15] Hajra KM, Chen DY, Fearon ER (2002). The SLUG zinc-finger protein represses E-cadherin in breast cancer. Cancer research.

[16] Olmeda D, Jorda M, Peinado H, Fabra A, Cano A (2007). Snail silencing effectively suppresses tumour growth and invasiveness. Oncogene.

[17] Fernandes AM, Valera FC, Anselmo-Lima WT (2008). Mechanism of action of glucocorticoids in nasal polyposis. Brazilian journal of otorhinolaryngology.

[18] Godoy P (2010). Dexamethasone-dependent versus -independent markers of epithelial to mesenchymal transition in primary hepatocytes. Biological chemistry.

[19] Sohal SS (2014). A randomized controlled trial of inhaled corticosteroids (ICS) on markers of epithelial-mesenchymal transition (EMT) in large airway samples in COPD: an exploratory proof of concept study. International journal of chronic obstructive pulmonary disease.

[20] Zhang YE (2009). Non-Smad pathways in TGF-beta signaling. Cell research.

[21] Ning W (2002). TGF-beta1 stimulates HO-1 via the p38 mitogen-activated protein kinase in A549 pulmonary epithelial cells. American journal of physiology. Lung cellular and molecular physiology.

[22] Xu J, Lamouille S, Derynck R (2009). TGF-β-induced epithelial to mesenchymal transition. Cell research.

[23] Pawliczak R, Lewandowska-Polak A, Kowalski ML (2005). Pathogenesis of nasal polyps: an update. Current allergy and asthma reports.

[24] Rinia AB, Kostamo K, Ebbens FA, van Drunen CM, Fokkens WJ (2007). Nasal polyposis: a cellular-based approach to answering questions. Allergy.

[25] Bachert C, Gevaert P, Holtappels G, Cuvelier C, van Cauwenberge P (2000). Nasal polyposis: from cytokines to growth. American journal of rhinology.

[26] Cho JS (2012). Epigenetic regulation of myofibroblast differentiation and extracellular matrix production in nasal polyp-derived fibroblasts. Clinical and experimental allergy: journal of the British Society for Allergy and Clinical Immunology.

[27] Konnecke, M. *et al*. Epithelial-Mesenchymal Transition in Chronic Rhinosinusitis: Differences Revealed Between Epithelial Cells from Nasal Polyps and Inferior Turbinates. *Archivum immunologiae et therapiae experimentalis*, doi:10.1007/s00005-016-0409-7 (2016). 10.1007/s00005-016-0409-7 27393708

[28] Meng J (2013). The development of nasal polyp disease involves early nasal mucosal inflammation and remodelling. PloS one.

[29] Kasai H, Allen JT, Mason RM, Kamimura T, Zhang Z (2005). TGF-beta1 induces human alveolar epithelial to mesenchymal cell transition (EMT). Respiratory research.

[30] Coutinho AE, Chapman KE (2011). The anti-inflammatory and immunosuppressive effects of glucocorticoids, recent developments and mechanistic insights. Molecular and cellular endocrinology.

[31] Tait AS, Butts CL, Sternberg EM (2008). The role of glucocorticoids and progestins in inflammatory, autoimmune, and infectious disease. Journal of leukocyte biology.

[32] Mygind N, Andersson M (2006). Topical glucocorticosteroids in rhinitis: clinical aspects. Acta oto-laryngologica.

[33] Zhang L, Lei W, Wang X, Tang Y, Song J (2010). Glucocorticoid induces mesenchymal-to-epithelial transition and inhibits TGF-beta1-induced epithelial-to-mesenchymal transition and cell migration. FEBS letters.

[34] Carayol N (2002). Regulation of E-cadherin expression by dexamethasone and tumour necrosis factor-alpha in nasal epithelium. The European respiratory journal.

[35] Doerner AM, Zuraw BL (2009). TGF-beta1 induced epithelial to mesenchymal transition (EMT) in human bronchial epithelial cells is enhanced by IL-1beta but not abrogated by corticosteroids. Respiratory research.

[36] Jang YH (2013). Effects of dexamethasone on the TGF-beta1-induced epithelial-to-mesenchymal transition in human peritoneal mesothelial cells. Lab Invest.

[37] Kolosova I, Nethery D, Kern JA (2011). Role of Smad2/3 and p38 MAP kinase in TGF-beta1-induced epithelial-mesenchymal transition of pulmonary epithelial cells. Journal of cellular physiology.

[38] Gui T, Sun Y, Shimokado A, Muragaki Y (2012). The Roles of Mitogen-Activated Protein Kinase Pathways in TGF-beta-Induced Epithelial-Mesenchymal Transition. Journal of signal transduction.

[39] Medici D, Hay ED, Olsen BR (2008). Snail and Slug promote epithelial-mesenchymal transition through beta-catenin-T-cell factor-4-dependent expression of transforming growth factor-beta3. Molecular biology of the cell.

[40] Aomatsu K (2011). TGF-beta induces sustained upregulation of SNAI1 and SNAI2 through Smad and non-Smad pathways in a human corneal epithelial cell line. Investigative ophthalmology & visual science.

[41] Bolte S, Cordelieres FP (2006). A guided tour into subcellular colocalization analysis in light microscopy. Journal of microscopy.

[42] Monsel A (2014). Analysis of autofluorescence in polymorphonuclear neutrophils: a new tool for early infection diagnosis. PloS one.

